# An Effective Model for Improving Global Health Nursing Competence

**DOI:** 10.3389/fpubh.2016.00191

**Published:** 2016-09-13

**Authors:** Sun-Joo Kang

**Affiliations:** ^1^School of Nursing, Cheju Halla University, Jeju-si, Republic of Korea

**Keywords:** nursing competence, global health nursing, program effectiveness, evaluation, culture understanding

## Abstract

This paper proposed an effective model for improving global health nursing competence among undergraduate students. A descriptive case study was conducted by evaluation of four implemented programs by the author. All programs were conducted with students majoring in nursing and healthcare, where the researcher was a program director, professor, or facilitator. These programs were analyzed in terms of students’ needs assessment, program design, and implementation and evaluation factors. The concept and composition of global nursing competence, identified within previous studies, were deemed appropriate in all of our programs. Program composition varied from curricular to extracurricular domains. During the implementation phase, some of the programs included non-Korean students to improve cultural diversity and overcome language barriers. Qualitative and quantitative surveys were conducted to assess program efficacy. Data triangulation from students’ reflective journals was examined. Additionally, students’ awareness regarding changes within global health nursing, improved critical thinking, cultural understanding, and global leadership skills were investigated pre- and post-program implementation. The importance of identifying students’ needs regarding global nursing competence when developing appropriate curricula is discussed.

## Introduction

Advances in medical technology, free traffic between countries, and burgeoning multicultural families provide new challenges to nursing education. In Korea, and in other countries, how to prepare students majoring in healthcare sciences has generated fervent discussion. Appropriate methods for training students to demonstrate appropriate attitudes regarding a culturally diverse landscape are needed (1–3).

Conceptualizing global health nursing has also been challenging. Archambault (4) outlined the following core factors: being a global citizen, social justice, equity in health, and health determinants. Subsequently, the Canadian Association of Graduate School of Public Health implemented research to identify core global health competencies, which include six categories: global burden of a disease; health implications of migration, travel, and displacement; social and environmental determinants of health; globalization of health and healthcare; healthcare in low-resource settings; and health as a human right and developmental resource. Previous research provides a useful guideline for improving global nursing competence within undergraduate nursing programs (3). Nursing education in the Republic of Korea developed during the pre-committee of nursing education accreditation in 2000. In 2011, the committee was designated as an accreditation board by the Ministry of Education. The committee has since been evaluating and accrediting 4-year bachelors in nursing programs and 3-year college programs. One of the educational objectives of a bachelor’s degree in nursing program is global nursing competency, and most programs strive to attain this goal through diverse efforts (5).

In other countries, several studies have clarified global health competency since the late 1990s (3, 5–9), and quantitative and qualitative data analytic methods have been implemented (10–12). Some work has even been done in South Korea since 2000 (13–16). Especially, four consecutive programs to increase undergraduate nursing students’ global nursing competency were provided by a university where the author works (14–18). However, few studies have done a student needs assessment before program operation and design. Thus, we have been limited in our ability to assess program efficacy both before and after its implementation (14–18).

On the other hand, the United Nations has enacted a resolution of outlining 17 sustainable development goals for global citizenry. This resolution is aimed at reducing health service inequalities while improving individuals’ overall health status (19). Prior to this resolution, the World Health Organization (WHO) recommended a global nursing education standard in 2010, and global nursing competence was included as a necessary core outcome (20, 21). Therefore, an effective global health nursing program model needs to include nursing competence for implementation, and the program model proposed by this research would contribute to preparing potential global nursing leaders undertaking nursing programs.

## Methods

### Research Design

This was a descriptive case study that analyzed global health and nursing programs, mostly Korean nursing students, for developing an effective program model regarding global nursing competency targeted toward undergraduate nursing students.

### Research Target

Global health and nursing programs implemented from 2013 to 2015 were the focus of our study. Four programs were used, and operated in an Asian country within the development stage and dealing with low healthcare resources.

### Data Collection

Information regarding the program’s plan and effect of each program were collected from published research papers on these programs (14–18).

### Data Analysis

All gathered data were analyzed for whether the programs assessed students’ needs, programs’ credit approval, program efficacy variables, and any other special characteristics regarding effectiveness.

## Results

### General Characteristics

The programs were implemented during an academic vacation of less than 2 weeks (see Table 1). Case 1 was held in Thailand to help improve global disaster responses (14) Case 2 was held in the Philippines for increasing global nursing leadership (15). Case 3 (16) and Case 4 (17) were operated in Vietnam to increase global assistance and leadership understanding. Participants’ majors varied from mostly nursing to other health care-related fields.

**Table 1:** **General program characteristics**.

	Purpose	Field training place	Student major
Case 1	International disaster response program	Caren village, Thailand	Nursing and paramedic
Case 2	Capacity building for global nursing leadership	Manila, Philippines	Nursing only
Case 3	Understanding improvement to international development cooperation	Hochimin and Binh Dinh province, Vietnam	Nursing and health-related majors
Case 4	Improvement to global nursing leadership	Hanoi and Hue city, Vietnam	Nursing and health-related majors

### Needs Assessment Results

Case 4 was the only program that assessed undergraduate students’ global health nursing competency between a present and desired level using a systematic tool (17). Case 4 then applied priorities based on differences in competency levels and developed and implemented a program. However, the other three programs (14–16) did not survey students’ needs prior to program operation, so no pre–post assessments were possible. For instance, Case 1 used the International Council of Nurses’ four disaster competencies to assess participants’ knowledge prior to program operation. Cases 2 and 3 pre-evaluated students’ critical thinking and global leadership abilities.

### Program Design

All four programs were designed to run for over 1 week. Program design was mainly conducted by nursing professors, with help from situ experts; however, only Case 4 considered six categories of global health nursing competency prior to designing the program (17). Case 1 focused on the effects of displacement and health as a human right related to global disaster responses (14). Cases 2 and 3 comprised health determinants within low-resource environments and examined healthcare globalization (15, 16). The scope of visitations from special organizations included the United Nations Economic and Social Commission for Asia and the Pacific (UNESCAP), the WHO Western-pacific regional office, *in situ* universities and affiliated hospitals, and elementary schools or other hospitals commissioned through official Korean developmental assistance (14–18). Participants commonly prepared case-relevant health education materials for children in all four programs (See Table 2).

**Table 2:** **Composition of the four programs**.

	Pre-learning	Program		
		Special lecture	Health education	Visitation
Case 1	5-case analysis for 5 weeks	Definition and types of disasters and refugees		UNESCAP^a^
		Ethical considerations of health issues for the dispatched population		Thailand–Burma Border Consortium Caren refugee village
		Community health assessment		
Case 2	Lectures, discussion presentation for a 2-day workshop	Tuberculosis project	Children in elementary school and low economic villages	WHO West-pacific regional office
		WHO^b^ activities		University of Philippines
		UN functions and roles		Community nursing practice
		Career development		
Case 3	2-credit course completion	Vietnam health care system	Children in elementary school, Binh dinh Hospital, and others	KOICA^c^
		UN roles and how to join		Hochimin and other universities
		Nursing career development		Hochimin Hospital
Case 4	2-credit course completion	Healthcare systems in Vietnam and Korea	Elementary school and nursing home residents in Hue city, along with other activities	KOICA Hanoi
		Global nursing leadership		Hue university
		Information technology		Hue general hospital
		Future direction of Vietnam Nurses Association (VNA)		Hue-Halla nursing education Center
		UN functions and roles		

*^a^United Nations Economic and Social Commission for Asia and the Pacific*.

*^b^World Health Organization*.

*^c^Korea International Cooperation Agency*.

### Program Implementation

Providing facilitation including monitoring and feedback while implementing a program, as well as considering program development, is essential for overseas implementation (See Table 3). For all programs, three to five professors guided participants with the help of five to six volunteers from each country (14–17). *In situ* experts took on a coordination role in order to address any unexpected changes during implementation, and participants were placed into groups of four or five in four programs (14–17). Except for Case 1, the other three programs invited one student to serve as a team member helper/interpreter to reduce any language barriers or cultural misconceptions. After the education component, groups had time to share what they learned. Students were better able to understand each member’s cultural differences, communalities, and how to communicate and cooperate.

**Table 3:** **Facilitation for program implementation**.

	Mentor	Group formation	Interpreter
Case 1	2 professors	4–5 participants/group	1 field expert from Thailand
	1 field expert		
Case 2	2 professors	4–5 participants/group	5 students from Vietnam
	1 field expert		
Case 3	5 Professors	4–5 participants/group	5 students from Philippines
Case 4	4 professors	4–5 participants/group	5 students from Vietnam
	1 field expert		

### Program Evaluation

Programs tried to identify any knowledge/attitude changes after program participation via a quantitative survey, along with qualitative methods employing group interviews and reflective journal assessments provided by the students (See Table 4). Case 1 used the *t*-test, whereas the other three programs’ effects were analyzed by paired *t*-tests. Cases 3 and 4 used a similar method, but Case 4 identified students’ needs assessment, while Case 3 did not assess participants’ needs prior to designing the program (14–18).

**Table 4:** **Evaluation methods of four programs**.

	Quantitative method		Qualitative method
	Survey tools	Measurement	
Case 1	Pre–post disaster competency survey using questionnaires developed by researchers (Cronbach’s α value 0.87)	*t*-test with Wilcoxon signed ranks	Content analysis of two additional open-ended questions followed by interview during the post-test
Case 2	Pre–post survey on critical thinking disposition and global leadership ability using Yoon and Song’s questionnaire (Cronbach’s α value 0.84 and 0.88, respectively)	Paired *t*-test	none
Case 3	Pre–post survey on critical thinking disposition and global leadership ability using Yoon and Song’s questionnaire (Cronbach’s α value 0.84 and 0.88, respectively)	Paired *t*-test	Content analysis of reflective journal written by participants
Case 4	Pre–post knowledge and attitude change survey using questionnaires developed Kang and Piao (Cronbach’s α value 0.87)	Paired *t*-test	Content analysis of reflective journal written by participants

The effect of each program was unique to the program’s intended outcomes. Case 1 showed significant knowledge change in disaster competency after the program (14), whereas Case 2 and Case 3 showed positive change in critical thinking disposition and global leadership ability after program participation (15, 16). Case 4 also showed a statistical difference in global health nursing knowledge after the program (18) (See Table 5).

**Table 5:** **Summary of meaningful results**.

	Pre-mean ± SD	Post-mean ± SD	Wilcoxon’s signed-rank test *Z*-value		Paired *t*-test	
			*z*	*p*	*t*	*p*
Case 1	2.18 ± 0.68	6.30 ± 0.84	−3.73	0.000		
Case 2	93.9 ± 11.5^a^	95.9 ± 12.5			−2.038	<0.043
	58.9 ± 10.0^b^	61.6 ± 9.2			−3.414	0.001
Case 3	101.12 ± 10.81^a^	103.82 ± 10.20			−4.000	0.001
	67.10 ± 7.45^b^	69.42 ± 7.36			−3.420	0.001
Case 4	21.58 ± 3.73	29.19 ± 8.40			−6.081	0.000

*^a^Critical thinking disposition*.

*^b^Global leadership ability*.

On the other hand, content analysis on the reflective journal between Case 3 and Case 4 differed in their sub-concepts and domains. In Case 3, 15 sub-concepts outlining global health, nursing, and capacity development emerged (16). However, Case 4 addressed competencies regarding global nursing, global leadership, and cultural understanding (18). This was possible because participants’ needs regarding global health nursing were pre-evaluated in Case 4. As such, Case 4 reflected more practical thinking of the participants.

## Discussion

Case 4 was the only program that assessed participants’ educational needs in order to do a pre- and post-implementation investigation for improving global health nursing competency. The other programs intended to provide relevant knowledge developing self-studies and workshops. Additionally, Case 4 operated as a one-semester course before *in situ* training. Overall, to accomplish objectives for global nursing competency education, it is necessary to create a fundamental and systematic plan for building curricula in accordance with students’ desired level of competency (18).

Hwang (22) recommended an educational program that was devised by analyzing trainees’ educational needs. Curtin (23) and Dirk (24) also argued the importance of assessing students’ needs. Considering the current status of global nursing programs, which are mostly provided as an extracurricular program rather than curricular program, it is more necessary to plan for the achievement of the program’s purpose within a very limited time duration.

During the design phase, all programs operated for 1 week within a one or two-semester student exchange program. This type of strategy helps to ease financial burden and forces implementers to be efficient with their program, which can help increase program efficacy. When designing a global health nursing program, global disease burden, health globalization, and social determinants were considered first based on previous research (9). Field experience was recommended for students from diverse social and cultural backgrounds in order to boost program effectiveness. All four cases within the current study included this within its program design.

The field experience component is a unique strategy that can help overcome language and cultural barriers during health education activities, special lectures, and group work discussions and presentations. For instance, Case 1 excluded health education for refugees because visitation to a refugee camp was strictly controlled (14). Thus, students conducted visits at refugee-related UN and non-governmental organizations, lectures, and discussions. Case 1 provided four instances where students could analyze and submit a case report. This was deemed as an important substitute to direct, *in situ* learning experiences (13, 14).

The other three programs (except Case 1) compared the effect of the program using paired *t*-tests and two of them added content analysis for further understanding of participants’ attitude change in global health nursing. It is highly recommended to plan a similar program to attain the objective of global nursing competency in 4-year nursing programs. Quantitative and qualitative methods were used to assess most of the cases. Quantitatively, assessments of changes in knowledge and attitudes were common; qualitative methods included group interviews, open-ended questions, and journal/diary reviews. Results from Case 4 are similar to those from Saiboon et al. (25) in terms of overseas field experience provisions for stimulating career advancement and facilitating global leadership, as well as understanding cultural diversity.

## Conclusion and Recommendation

In conclusion, what would be an effective model for improving global nursing competency programs for undergraduate students? It is very useful to identity the benefits and limitations of analyzed programs in this research. As shown in Table 6, during the first assessment phase, credit-based or non-credit-based formats need to be considered. Previous knowledge should also be examined in order to engage student interest. A baseline needs assessment should follow so as to adequately accomplish the nursing program’s educational objectives. Finally, a training site needs to be decided upon and additional factors (e.g., finances, route, and program content) need to be determined.

**Table 6:** **Effective model to design a program for global nursing competency**.

Assessment	Design	Implementation	Evaluation
Open course	Participant selection	Mixed-nationality participants Writing a reflective journal	Analyzing reflective journals
Pre-survey needs assessment	Program composition according to the survey results	Group activities: community health assessment, visitation to low-resource health institutions, and health education	Debriefing and presentation post-survey
Site selection	Expert review	Monitoring activities	Feedback

During the second phase (design), program content is developed based on the prior needs assessment and comments from field experts. Participant selection should then proceed by considering requirements and course objectives. If participants are “global citizens,” sustainable developmental goals for health equality or any effort therein could maximize the field experience.

In the implementation phase, grouping participants should help promote better understanding of the healthcare and cultural environment, providing a more mutually dynamic learning experience. Furthermore, real-time monitoring and feedback from staff, professors, and field experts can aid program efficacy.

To evaluate the program’s effectiveness, both quantitative and qualitative methods are necessary to determine program effectiveness, as well as changes to knowledge and attitudes among participants. However, evaluations of participants’ critical thinking and leadership abilities may be somewhat inappropriate when addressing program effectiveness. This is because most participants embody these traits more than the average student.

The present study outlined an effective model for improving global health nursing competency. Based on needs assessment pre-survey, specific variables representing program effectiveness should be scrutinized to better promote a program’s objectives.

A few program limitations should be noted. For instance, only a small number of students could reasonably participate due to financial strains and the ability to select an appropriate field site. Thus, table-top case simulations and field program briefing sessions may be needed to provide indirect learning experiences. Nevertheless, both direct and indirect methods could have immense utility for facilitating global health nursing competency.

## Author Contributions

The author confirms being the sole contributor of this work and approved it for publication.

## Conflict of Interest Statement

The author declares that the research was conducted in the absence of any commercial or financial relationships that could be construed as a potential conflict of interest.
